# Hydrogen Production via Electrolysis of Wastewater

**DOI:** 10.3390/nano14070567

**Published:** 2024-03-25

**Authors:** Lijun Huang, Chaoqiong Fang, Ting Pan, Qigang Zhu, Tiangeng Geng, Guixiang Li, Xiao Li, Jiayuan Yu

**Affiliations:** 1 Institute for Advanced Interdisciplinary Research (iAIR), School of Chemistry and Chemical Engineering, University of Jinan, Jinan 250022, China; 2 Zhejiang Hehui Ecological Environment Technology Co., Ltd., Jiaxing 314201, China; 3 Institute of Chemical Sciences and Engineering, École Polytechnique Fédérale de Lausanne (EPFL), CH-1015 Lausanne, Switzerland; 4 Zhejiang Hehui Sludge Disposal Co., Ltd., Jiaxing 314201, China; 5 School of Chemical Engineering and Technology, Tianjin University, Tianjin 300072, China

**Keywords:** hydrogen production, degradation of pollutants, waste reforming, electrocatalysis, photoelectrochemistry

## Abstract

The high energy consumption of traditional water splitting to produce hydrogen is mainly due to complex oxygen evolution reaction (OER), where low-economic-value O_2_ gas is generated. Meanwhile, cogeneration of H_2_ and O_2_ may result in the formation of an explosive H_2_/O_2_ gas mixture due to gas crossover. Considering these factors, a favorable anodic oxidation reaction is employed to replace OER, which not only reduces the voltage for H_2_ production at the cathode and avoids H_2_/O_2_ gas mixture but also generates value-added products at the anode. In recent years, this innovative strategy that combines anodic oxidation for H_2_ production has received intensive attention in the field of electrocatalysis. In this review, the latest research progress of a coupled hydrogen production system with pollutant degradation/upgrading is systematically introduced. Firstly, wastewater purification via anodic reaction, which produces free radicals instead of OER for pollutant degradation, is systematically presented. Then, the coupled system that allows for pollutant refining into high-value-added products combined with hydrogen production is displayed. Thirdly, the photoelectrical system for pollutant degradation and upgrade are briefly introduced. Finally, this review also discusses the challenges and future perspectives of this coupled system.

## 1. Introduction

With the development of industrialization, energy consumption is increasing. Massive use of non-renewable resources, such as fossil fuels, has brought a serious energy crisis and environmental pollution. Therefore, it is crucial to seek clean, efficient, and environmentally friendly energy alternatives. Hydrogen energy, regarded as the key to the future of green energy, has received widespread attention due to its zero-pollution, high-energy, and resource-rich properties [1,2]. Nowadays, hydrogen is still mainly produced by reforming fossil energy represented by coal and natural gas. However, this process causes severe environmental pollution and excessive energy consumption. Hydrogen production through water splitting driven by renewable energy, which does not cause environmental pollution problems, such as carbon emissions, is an important way to produce green hydrogen [3,4,5,6,7,8,9]. Water electrolysis technology usually includes hydrogen evolution reaction (HER) [10,11,12] and oxygen evolution reaction (OER) [13,14,15]. The OER involves a four-electron transfer process, resulting in slow reaction kinetics and high overpotential. Therefore, a large amount of electric power needs to be consumed to drive the reaction. The high overpotential of OER causes the actual water decomposition voltage to be higher than the theoretical voltage of 1.23 V. Therefore, the high energy consumption is a key limiting factor for the foreground of hydrogen production via water electrolysis.

In traditional overall water splitting, the anode product is a low-value commodity. What is more, hydrogen and oxygen produced at the same time present a potential explosion risk, so a chamber must be employed to separate the cathode and anode. According to recent studies [16], the use of thermodynamically more favorable reactions at the anode instead of the OER, coupled with cathodic HER, can increase hydrogen production efficiency and reduce voltage energy consumption (Table 1).

**Figure 1:**
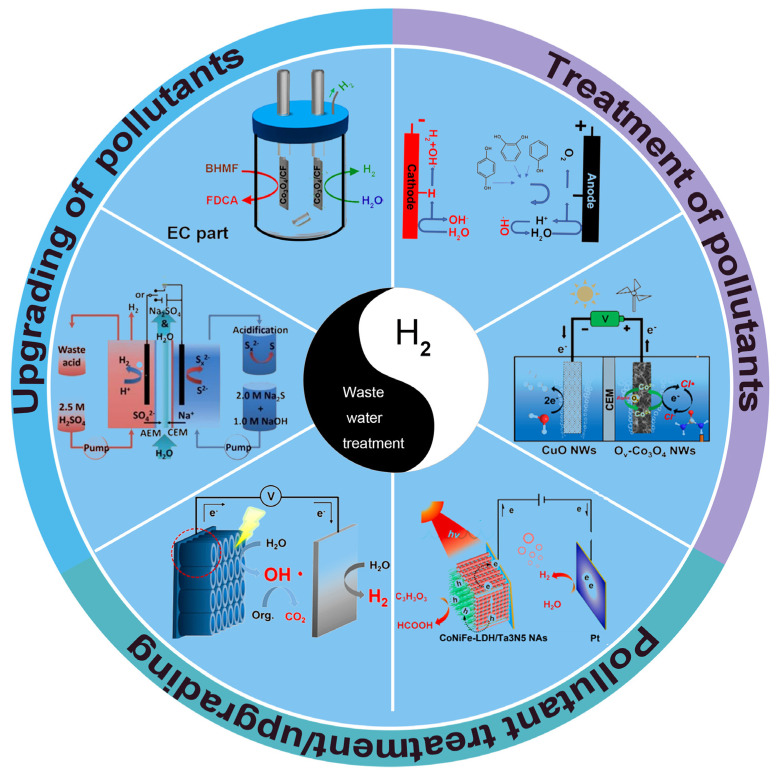
Different hybrid systems for coupling hydrogen generation with wastewater treatment. Ref. [17] Copyright 2023, Elsevier. Ref. [18] Copyright 2021, Wiley-VCH. Ref. [19] Copyright 2022, Elsevier. Ref. [20] Copyright 2021, American Chemical Society. Ref. [21] Copyright 2017, American Chemical Society. Ref. [22] Copyright 2021, Elsevier.

The electrochemical hydrogen generation system combined with a non-OER process has attracted much attention. Also, some recent articles have summarized the coupled system, such as biomass oxidation/organic small molecule oxidation coupled hydrogen production. However, a critical review focused on wastewater purification/pollutant refining coupled with hydrogen production has not been reported. In this review (Figure 1), wastewater purification coupled with hydrogen production is firstly presented. Subsequently, a pollutant refining coupled hydrogen production system is introduced. Finally, the problems faced in research related to hydrogen production coupled with pollutant transformation through electrocatalytic technology are summarized, and an outlook for future development is also presented.

**Table 1:** Performance comparison between the traditional electrolytic water system and some of the new coupled systems. Type I: Overall water splitting system. Type II: Hydrogen generation coupled with water purification system. Type III: Hydrogen generation coupled with waste reforming system.

Type of Cell	Cathodic Catalyst	Anodic Catalyst	Driving Voltage	Pollutants in Wastewater	Product	Ref.
Type I	Co-Mo_2_C	Co-Mo_2_C	1.68 V (10 mA cm^−2^)	-	H_2_+O_2_	[23]
	CoBO_x_/NiSe	CoBO_x_/NiSe	1.48 V (10 mA cm^−2^)	-	H_2_+O_2_	[24]
	Ni_5_P_4_@NiCo_2_O_4_	Ni_5_P_4_@NiCo_2_O_4_	1.65 V (100 mA cm^−2^)	-	H_2_+O_2_	[25]
	Ni/Ni(OH)_2_	Ni/Ni(OH)_2_	1.59 V (10 mA cm^−2^)	-	H_2_+O_2_	[26]
	CoMoP/Ni_3_S_2_	CoMoP/Ni_3_S_2_	1.54 V (10 mA cm^−2^)	-	H_2_+O_2_	[27]
	(Ni-Fe)S_x_/NiFe(OH)_y_	(Ni-Fe)S_x_/NiFe(OH)_y_	1.46 V (10 mA cm^−2^)	-	H_2_+O_2_	[28]
	HOF-Co_0.5_Fe_0.5_/NF	HOF-Co_0.5_Fe_0.5_/NF	1.36 V (10 mA cm^−2^)	-	H_2_+O_2_	[29]
	NiCo foam	NiFe foam	1.52 V (10 mA cm^−2^)	-	H_2_+O_2_	[30]
	Mo–NiCoP	E-Mo–NiCoP	1.61 V (10 mA cm^−2^)	-	H_2_+O_2_	[31]
	F_0.25_C_1_CH/NF	F_0.25_C_1_CH/NF	1.45 V	-	H_2_+O_2_	[32]
Type II	CoNi@CN-CoNiMoO)	CoNi@CN-CoNiMoO)	1.58 V (500mA cm^−2^)	Urea	H_2_+NO_2_	[33]
	Fe@N-CNT/IF	IF	1.09 V (20mA cm^−2^)	Organic and heavy metal	H_2_	[34]
	CoP/TiM	CoP/TiM	0.20 V (10mA cm^−2^)	Hydrazine	H_2_	[35]
	Cu(II)-Co_3_O_4_ NWs	Cu(II)-Co_3_O_4_ NWs	-	Urea	H_2_+NO_2_	[17]
	CuO	Ov-Co_3_O_4_	-	Urea	H_2_+NO_2_	[36]
Type III	CoO@C/MXene/NF	CoS_2_@C/MXene/NF	0.97 V	Sulfion	H_2_+S	[37]
	CoNi@NG	CoNi@NG	0.25 V	Sulfion	H_2_+S	[38]
	CC@N-CoP	CC@N-CoP	0.89 V (10 mA cm^−2^)	Sulfion	H_2_+S	[39]
	WS_2_	WS_2_	0.53 V	Sulfion	H_2_+S	[18]
	PdBi/NF	PdBi/NF	1.02 V	Alcohol	H_2_+3-Hp	[40]
	Co_3_O_4_/CF	Co_3_O_4_/CF	1.39 V (10 mA cm^−2^)	2,5-bis(hydroxymethyl)furan	H_2_+FDCA	[19]
	Pt/C	Cu(OH)_2_/NF	0.92 V (100 mA cm^−2^)	Glucose	H_2_+CHOCOOH	[41]
	Pt/C	Cu-Cu_2_O/CC	0.59 V (10 mA cm^−2^)	Glycerol	H_2_+HCOOH	[42]
	CoNi_0.25_P/NF	CoNi_0.25_P/NF	1.80 V (500 mA cm^−2^)	Polyethylene terephthalate	H_2_+HCOOH	[43]
	W-Ni_3_N/NF	N-Cu/Cu_2+1_O/CF	0.42 V (500 mA cm^−2^)	Formaldehyde	H_2_+HCOOH	[44]

## 2. Overall Water Splitting for Hydrogen Production

**Figure 2:**
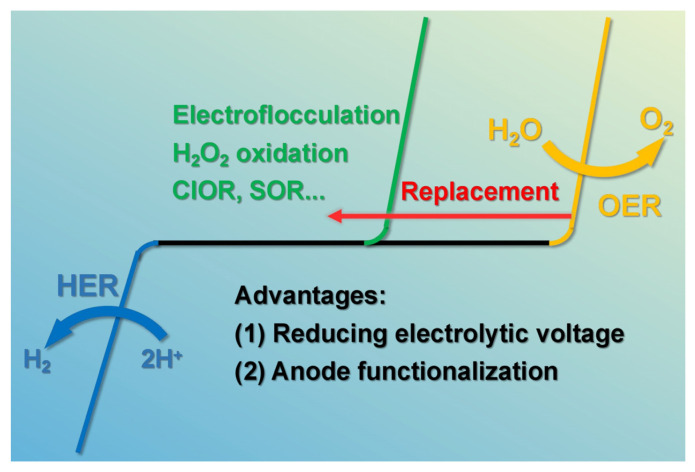
Illustration of the advantages of the hybrid electrolytic systems for replacing OER with other oxidation reactions.

Hydrogen production through water electrolysis is a promising technology that can convert distributed energy into hydrogen energy that can be stored. The design and development of catalysts with high activity are the key to realizing hydrogen production through water electrolysis. Recently, with the progress of nanotechnology (heterojunction engineering, doping engineering, defect engineering, etc.) for regulating the activity of electrocatalysts, a series of promising electrocatalysts have been developed. For instance, many transition metal-based compounds (Mo_3_Se_4_-NiSe [1], Co-Mo_2_C [23], Ni_5_P_4_@NiCo_2_O_4_ [25], CoMoP/Ni_3_S_2_ [27], Mo-NiCoP [31], etc.) with excellent performance become potential candidates as non-noble metal electrocatalysts for electrolysis of water. However, the theoretical decomposition water voltage of 1.23 V cannot be broken by regulating the activity of the catalyst. Therefore, the researchers propose hybrid water decomposition systems, which are introduced in the following part (Figure 2).

## 3. Electrocatalytic Hydrogen Production Coupled with Pollutant Removal

Industrial wastewater contains a lot of toxic and harmful pollutants, and most of them are high in salt (a total dissolved solid mass concentration of more than 3.5% and salt content of more than 1%) [45]. Traditional biological methods have been unable to effectively treat high-salt wastewater [46]. Electrochemical anodization to produce oxidizing free radicals (·OH, ·Cl) with strong oxidation capacity is an effective means to degrade pollutants. As mentioned earlier, traditional anodes for electrolyzing water produce low-value-added oxygen. Therefore, the coupled system for producing hydrogen at the cathode and water purification at the anode can expand the function of the electrolyzer [47].

**Figure 3:**
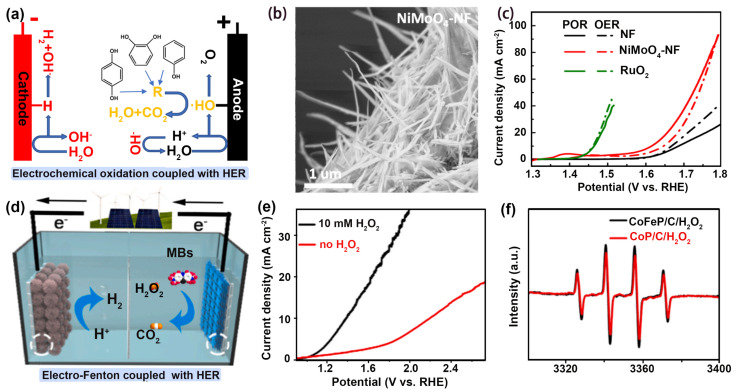
(**a**) Schematic diagram of dual-function electrolytic cell. (**b**) SEM image of NiMoO_4_-NF. (**c**) LSV curves of NF, NiMoO_4_-NF, and RuO_2_ for POR and OER. Ref. [20] Copyright 2021, American Chemical Society. (**d**) Schematic diagram of electro-fenton coupled with H_2_ production. (**e**) Polarization curves of CoFeP/C with/without 10 mM H_2_O_2_. (**f**) EPR spectra of radical species using CoFeP/C/H_2_O_2_ and CoP/C/H_2_O_2_ Ref. [53] Copyright 2021, Elsevier.

Hydroxyl radical (·OH) with the oxidation potential of 2.8 V is an important reactive oxygen species (ROS) that can efficiently degrade pollutants [48,49,50,51,52]. Efficient production of ·OH is the key to achieving synchronous hydrogen production and water purification. For instance, Mao et al. prepared NiMoO_4_ loaded on a nickel foam (NiMoO_4_-NF) anode catalyst using the hydrothermal method. The NiMoO_4_-NF anode can generate ·OH efficiently for phenol degradation (Figure 3a) [20]. The NiMoO_4_-NF layer was porous (Figure 3b), which not only facilitated the efficient formation of ·OH but was also conducive to the diffusion and enrichment of pollutants. As shown in Figure 3c, the performance of NiMoO_4_-NF was promoted through the addition of phenol, with the overpotential reduced from 410 to 380 mV at 10 mA cm^−2^. The stepping down of overpotential may be due to the high-efficiency degradation of phenol by ·OH. The high-efficiency anode reaction coupled with cathode hydrogen production was used to construct a hybrid electrolytic cell. On account of the operation condition and reaction parameters, the input electrical energy of this system only needed as low as 56.5 kWh (kg H_2_)^−1^.

To further reduce the drive voltage for the coupled system, researchers oxidized H_2_O_2_ to obtain ·OH to achieve the reduction of the driving voltage. For example, Tao et al. synthesized a needle-like CoFeP/C anode catalyst via hydrothermal and post-phosphorization processes, which was employed for the oxidation of H_2_O_2_ to produce ·OH (Figure 3d) [53]. Figure 3e indicates that H_2_O_2_ oxidation to produce ·OH is more favorable than OER. According to the electron paramagnetic resonance (EPR) spectroscopy (Figure 3f), Fe could optimize the electronic structure of CoP/C so as to oxidize H_2_O_2_ more efficiently to produce ·OH. In the CoFeP/C//CoP/C coupled system, MB was almost completely degraded after 40 min. The CoP/C cathode also possesses excellent HER performance, with the overpotential of 42.1 mV (vs. RHE) to achieve 10 mA cm^−2^. Due to highly active CoP/C and CoFeP/C, the flow system for the synchronization of H_2_ production and pollutant degradation just needed a voltage of 1.68 V.

**Figure 4:**
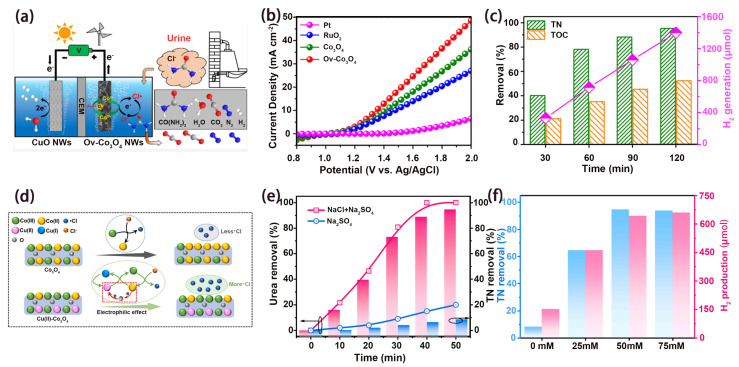
(**a**) Schematic illustration of ·Cl-mediated urea degradation and H_2_ production in O_v_-Co_3_O_4_ NWs and CuO NWs system. (**b**) LSV curves of different samples in 0.5 M NaCl. (**c**) TN/TOC removal and H_2_ generation while degrading actual urine wastewater. Ref. [36] Copyright 2022, American Chemical Society. (**d**) Schematic diagram of enhancement mechanism for ·Cl generation. (**e**) Urea and TN removal with/without 50 mM of NaCl using Cu(II)-Co_3_O_4_ NWs. (**f**) The performances of TN removal and H_2_ production at different Cl^-^ concentrations. Ref. [17] Copyright 2023, Elsevier.

Besides ·OH, chlorine radicals (·Cl) are also widely used to degrade pollutants due to their high activity and selectivity [54,55]. Urine is an abundant biological pollutant enriched with 80% nitrogen in domestic wastewater. Direct urine oxidation has sluggish kinetics owing to the complex six-electron transfer process [56,57,58]. As an alternative, urea degradation can be effectively achieved via ·Cl oxidation. For example, Zhang et al. prepared an Ov-Co_3_O_4_ NWs anode using hydrothermal and reduction methods, which was used for an anode urea degradation coupled hydrogen production system (Figure 4a) [36]. EPR spectroscopy showed that abundant Ov existed in Ov-Co_3_O_4_ NWs. Ov could accelerate the conversion between Co(II) and Co(III), which had a high redox potential and could activate Cl^-^ to ·Cl, thus effectively promoting urea degradation. In Figure 4b, O_V_-C_O3_O_4_ possessed the highest activity in all samples, which could produce the most ·Cl. In the coupled system, the removal rate of TN reached 97% in 2 h (Figure 4c), and the yield of H_2_ was 1390 μmol. Similarly, in order to increase the proportion of Co(III) in the catalyst, Xie et al. prepared Cu(II)-modified Co_3_O_4_ nanowires (Cu(II)-Co_3_O_4_ NWs) through simple hydrothermal and calcination methods for TN removal and H_2_ generation during urea degradation (Figure 4d) [17]. Based on the XPS spectra, it could be seen that the content of Co(III) in Cu(II)-Co_3_O_4_ (46.7%) was higher than that in Co_3_O_4_ (38.6%), and Cu could promote the formation of Co(III). As shown in Figure 4e, the chlorine-evolution-reaction-generated ·Cl could effectively promote urea degradation. The increase in chloride concentration was conducive to urea degradation (Figure 4f). TN removal and H_2_ generation on Cu(II)-Co_3_O_4_ achieved 94.7% and 642.1 μmol, respectively. Due to the simultaneous production of valuable hydrogen in the urea removal process, the treatment cost is lower than reverse osmosis and air-stripping.

**Figure 5:**
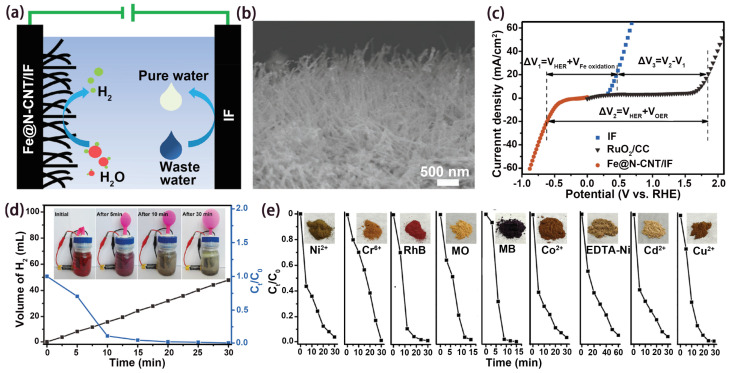
(**a**) Concept of cathodic HER and anodic water purification coupled system. (**b**) FESEM image of Fe@N-CNT/IF. (**c**) Polarization curve in 0.5 M of Na_2_SO_4_. (**d**) H_2_ yield and RhB removal rate of the coupled system driven by a 1.5 V AA commercial battery. (**e**) Purification performance of different pollutants. Ref. [34] Copyright 2019, Wiley-VCH.

In addition to the above methods of treating pollutants using free radicals, electroflocculation has the advantages of removing a wide range of pollutants and applying to a wide range of pH [59,60,61,62]. Our group reported a hybrid electrolysis system of hydrogen production at a cathode and electroflocculation-adsorbed pollutants at an anode through a hybrid electrolytic cell (Fe@N-CNT/IF(−))//IF(+) (Figure 5a) [34]. As can be seen from Figure 5b, Fe@N-CNT arrays covered the IF surface to form a multidimensional interfacial structure, which facilitated electron transfer and provided more electrocatalytic regions. As shown in Figure 5c, the cell voltage of the coupled system was 1.31 V lower than that of the overall water splitting at the current density of 20 mA cm^−2^. Because of the ultralow electrolysis voltage, only a 1.5 V battery could drive the coupled system for H_2_ generation and wastewater treatment (Figure 5d). For the homemade coupled system device, the generation rate of H_2_ was 4 mL min^−1^ at 1.5 V, and the degradation rate of Rhodamine B contained in electrolyte achieved 99.2% in 10 min. In addition, anodic flocculation also showed good results for the removal of various pollutants and heavy metal ions during 30 min (Figure 5e).

Thus, anodic wastewater treatment coupled with cathodic hydrogen production is a new system for energy conversion and pollutant removal simultaneously, and it improved the efficiency of wastewater treatment and reduced energy consumption. It has important application value and practical significance for the development of hydrogen energy utilization using wastewater as a resource.

## 4. Electrocatalytic Hydrogen Production Coupled with Pollutant Upgrade

The pollutants in the water body contain abundant organic (glycerin, glycol, methanol, formaldehyde) and inorganic elements (NO_3_^−^, NO_2_^−^, S^2−^, phosphate), which are potential resources. For instance, formaldehyde can be electrooxidized to formic acid, and S^2-^ can be electrooxidized to solid sulfur. Although water purification coupled with hydrogen production has realized the functionalization of the electrolyzer, the pollutants in the wastewater have not been fully utilized. If the anode reaction can be functionalized at the same time, upgrading pollutants into high-value products is a more promising way.

**Figure 6:**
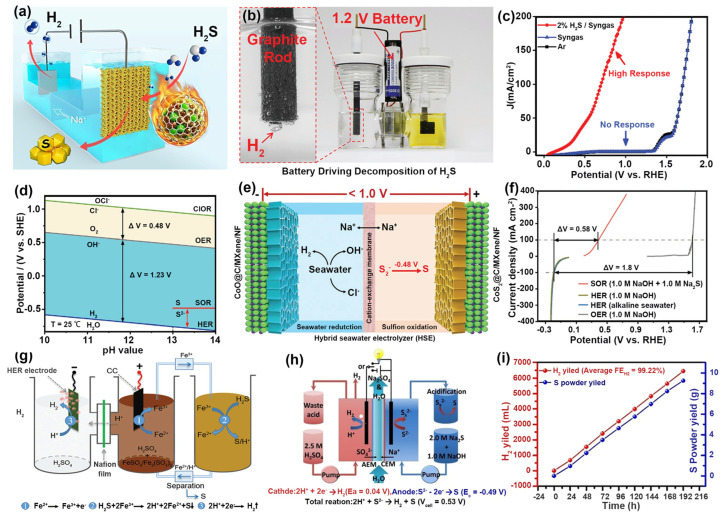
(**a**) Schematic diagram of H_2_ production and H_2_S decomposition. (**b**) The decomposition of H_2_S driven by a 1.2 V battery. (**c**) LSV curves of CoNi@NGs under different atmospheres. Ref. [38] Copyright 2019, Royal Society of Chemistry. (**d**) Pourbaix plot of SOR, HER, OER, and ClOR. (**e**) Schematic diagram of hybrid seawater electrolyzer. (**f**) Voltage difference of HER, SOR, and OER in different electrolytes. Ref. [37] Copyright 2021, Wiley-VCH. (**g**) Conceptual schematic plot of coupled system of HER and SOR. Ref. [39] Copyright 2018, Wiley-VCH. (**h**) Diagram of H_2_ generation flow cell coupled with SOR. (**i**) H_2_ and sulfur yield in electrolysis process. Ref. [18] Copyright 2021, Wiley-VCH.

Recently, with the rapid development of the industry, dyestuffs, pharmaceuticals, pesticides, petrochemicals, and other basic industries, a large amount of sulfide-containing wastewater has been discharged [63,64]. For example, the use of lye to absorb H_2_S in syngas can produce a large amount of sulfide-containing wastewater. Sulfur ions can be electrooxidized to (solid) sulfur (SOR, S^2−^ − 2e^−^ = S, −0.48 V vs. RHE) at a low potential, which is a promising treatment method for degrading wastewater and further obtaining the additional product of monosulfur. Currently, many works have utilized SOR instead of OER for simultaneous hydrogen production at low voltage and for the recovery of sulfur [37,38,39]. For example, Deng et al. proposed a templating assisted method to prepare non-precious CoNi nanoalloys encapsulated in nitrogen-doped graphene (CoNi@NGs) as an effective electrocatalyst for generating H_2_ and S simultaneously from sulfide-containing wastewater (Figure 6a) [38]. An HRTEM image confirmed that the metal NPs were fully encapsulated by a nitrogen-doped graphene shell. This structure protected the metal from corrosion and kept the catalyst highly active. Consequently, CoNi@NGs showed only 0.25 V onset potential for SOR. Furthermore, CoNi@NGs had outstanding stability over 500 h and 98% faradaic efficiency (FE) to H_2_ generation. Only a 1.2 V battery could actuate H_2_S decomposition (Figure 6b). The catalyst showed excellent selective removal performance of H_2_S in syngas (Figure 6c). DFT calculations confirmed that nitrogen doping synergistically with encapsulated metal alloys optimized the polysulfide intermediates’ adsorption on the graphene surface and enhanced SOR activity.

Given the current scarcity of fresh water, seawater stores almost unlimited amounts of hydrogen, accounting for 96.5 percent of the planet’s total water resources [65,66,67]. At the anode, the chlorine electrooxidation reaction (ClOR) not only competes with the OER to reduce energy efficiency, but also the oxidation potential of SOR can be reduced by 1.3–1.4 V compared with ClOR (Figure 6d). It offers the opportunity for seawater electrolysis to fully avoid harmful chlorination while significantly reducing energy costs. Qiu et al. synthesized CoO/MXene cathode catalysts and CoS_2_/MXene anode catalysts through hydrothermal and calcination methods [37]. These catalysts could be used for simultaneous sulfur and H_2_ production in a mixed seawater SOR and HER coupled system (Figure 6e). The catalyst was a nanoarray rough surface, and the structure could maximize the exposure of the active site and improve the catalytic activity. Therefore, based on anodic SOR coupled with a cathode HER system, the battery voltage could be reduced by two to three times compared with the alkaline overall water-splitting (OWS) reaction (Figure 6f). The rate of hydrogen generation was 5.34 mol h^−1^ g_cat_^−1^ at 300 mA cm^−2^ with stabilized seawater electrolysis for 180 h. Rapid oxidation of S^2−^ to sulfur was also achieved with a degradation efficiency of 80%.

The electrolyte environment also has an impact on the catalytic reaction kinetics. Generally speaking, the kinetics of HER are faster in acidic environments [68]. Zhou et al. prepared N-doped CoP as an electrocatalyst through hydrothermal and phosphoric calcination on a carbon cloth substrate [39]. The catalyst achieved hydrogen production and recovery of sulfur solids at low power consumption with the help of the Fe^2+^/Fe^3+^ redox reaction in an acidic medium (Figure 6g). XPS indicated that nitrogen doping reduced the D-band of CoP and weakened the adsorption of H on the CoP surface. It was beneficial to improve the HER performance. In an acidic media electrolyzer, only 42 mV was needed to achieve 10 mA cm^−2^ for HER, and the average FE could be achieved at 95.7% at different current densities. And, sulfur production efficiency was about 95.1%.

In acidic media, HER kinetics are more favorable, while S^2-^ is more easily oxidized in alkaline media to generate electrons. Therefore, designing a mixed-solution electrolyzer system for HER coupled with SOR can be more effective for improving efficiency and reducing energy consumption. Li et al. synthesized a WS_2_ nanosheet using a low-temperature molten salt-assisted process [18]. The catalyst was used in an anodic (alkaline SOR) and cathodic (acidic HER) coupled system (Figure 6h). The HRTEM showed the presence of a large number of edge dislocations, which could effectively promote the electrocatalytic activity. The coupled system could achieve 8.54 mA cm^−2^ at a bias-free voltage to produce H_2_ and degrade sulfide simultaneously. The H_2_ generation rate reached 336.3 L h^−1^ m^−2^ with FE_H2_ of 99.22% (Figure 6i). The alkali–acid hybrid electrochemical device had good stability.

**Figure 7:**
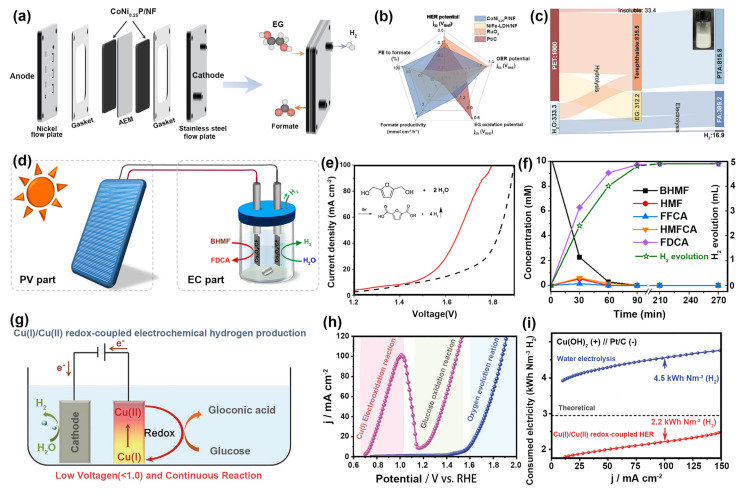
(**a**) Membrane electrode assembly pairs HER(−)//EG oxidation(+). (**b**) Comparison of cata-lytic performance of CoNi_0.25_P/NF. (**c**) Sankey diagram of PET upcycling quality process. Ref. [43] Copyright 2021, Nature Publishing Group. (**d**) Schematic diagram of PVEC with simultaneous oxidation of HMF and HER. (**e**) LSV curves in the absence and presence of 10 mM of BHMF. (**f**) Simultaneous oxidation and hydrogen evolution performance of HMF at 1.65 V voltage. Ref. [19] Copyright 2022, Elsevier. (**g**) Cu(I)/Cu(II) redox and electrochemical H_2_ production. (**h**) LSV curves of Cu(OH)_2_ electrodes. (**i**) Electricity consumption for H_2_ production of Cu(I)/Cu(II) redox-coupled HER and water electrolysis. Ref. [41] Copyright 2021, Wiley-VCH.

In recent years, the electrochemical oxidation of organics (such as ethylene glycol (EG) [69,70], 2,5-bis(hydroxymethyl)furan (BHMF), glucose [71], formaldehyde, polycyclic aromatic hydrocarbons, benzene series) derived from wastewater to generate value-added products has attracted great interest because it can achieve the refining of pollutants and H_2_ production at the same time [72,73,74,75,76,77,78,79,80]. Zhou et al. synthesized nickel foam (NF)-loaded Co and Ni phosphide bifunctional catalysts through electrodeposition and phosphide calcination methods for an EG oxidation-coupled hydrogen production system (Figure 7a) [43]. The HRTEM revealed that small Ni_2_P nanoparticles were inter-connected with CoP particles, forming CoP-Ni_2_P heterojunctions in CoNi_0.25_P. As shown in Figure 7b, high current density (500 mA cm^−2^) and formic acid FE (80%) were achieved at low voltage (1.8 V). In addition, the recycling of PTA monomer to produce high-value-added products (KDF and hydrogen) could be realized from PET plastic (Figure 7c). This work provided direction for the upgrading of plastic waste and the preparation of high-value-added commodity chemicals and hydrogen. Similarly, Chen et al. prepared hydrangea-like Co_3_O_4_ on cobalt foam (Co_3_O_4_/CF) as a bifunctional electrocatalyst to integrate HMF oxidation reactions (HMFOR) and HER (Figure 7d) [19]. The HRTEM image showed the existence of nanopores, channels, and grain boundaries in the structure, which was rich in electroactive sites to promote HMFOR (Figure 6e). As a result, the Co_3_O_4_ catalyst achieved the conversion of HMF to FDCA up to 93.2% and 99.8% FE_H2_ (Figure 7f). Finally, the commercial solar cell was connected in parallel with a symmetric filmless electrolyzer to achieve FDCA and H_2_ production in natural sunlight.

In addition to using the oxidation of organics to replace the OER to lower the reaction potential, the anode itself undergoes oxidation that can additionally lower the potential further. Zhang et al. prepared Cu(OH)_2_ arrays with 3D structures grown on copper foam and developed a Cu(I)/Cu(II) redox system for H_2_ generation using glucose as a reducible sacrifice agent in Figure 7g [41]. The Cu(OH)_2_ produced through electrooxidation could be reduced to Cu_2_O instantly by glucose under anodic oxidation conditions, resulting in the redox cycle (Figure 7h). It only required 0.92 V to reach 100 mA cm^−2^ (Figure 7i). Moreover, the electricity consumption was 2.2 kWh Nm^−3^ (H_2_), which is much lower than that for conventional water electrolysis (4.5 kWh Nm^−3^ (H_2_)). The system significantly reduces energy consumption.

## 5. Photo-Electrocatalytic Hydrogen Production Coupled with Pollutant Removal/Upgrade

The photoelectrochemical (PEC) method is a method of combining external potential bias with light to separate carriers into cathodes and anodes [81,82]. Under illumination, the semiconductor electrode can produce ROS with strong oxidation. It is widely used in various photochemical transformations containing pollutants removal, ROS production, and the reduction of proton/water to molecular hydrogen [83,84,85,86,87,88,89,90,91,92,93,94,95,96,97,98].

**Figure 8:**
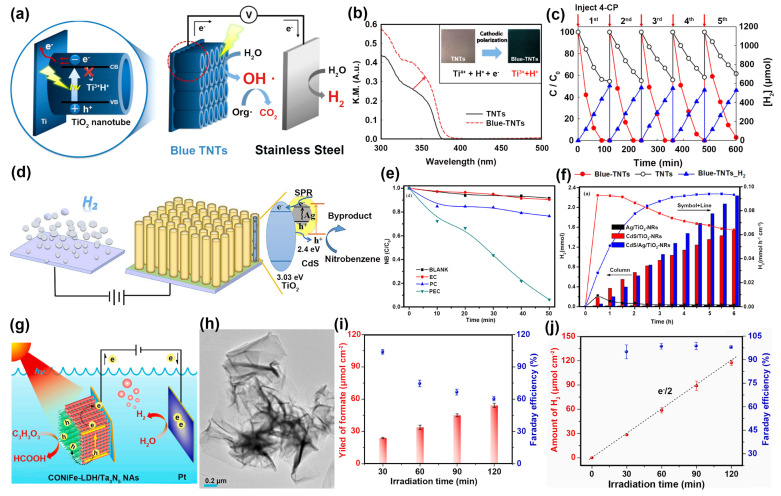
(**a**) Schematic diagram of photoelectric chemical water treatment. (**b**) UV-Vis of TNTs and blue-TNTs. (**c**) The stability curves of PEC 4-CP degradation and H_2_ generation. Ref. [21] Copyright 2017, American Chemical Society. (**d**) Possible mechanism diagram of H_2_ production. (**e**) Comparison of catalytic performance of CdS/Ag/TiO_2_-NRs in different conditions. (**f**) Comparison of H_2_ production performance on different catalysts. Ref. [106] Copyright 2019, Elsevier. (**g**) Schematic diagram of solar-driven GOR coupled to HER. (**h**) TEM images of CoNiFe-LDHs. (**i**) Yield of formate and FE_formate_. (**j**) Amount of H_2_ and FE_H2_. Ref. [22] Copyright 2021, Elsevier.

TiO_2_ has the advantages of high catalytic activity, stability, non-toxicity, low price, and easy regeneration, which make it a promising semiconductor catalyst widely used in photocatalytic systems [99,100,101,102]. However, TiO_2_ has a wide forbidden bandwidth (3.23 eV) and can only be excited by ultraviolet light with a wavelength of less than 387 nm to produce photocatalytic activity. However, the energy of UV light accounts for a small percentage of the solar spectrum, so the development of TiO_2_ applications is limited. To overcome the abovementioned shortcoming, the absorption range has been broadened by constructing defects, forming heterojunctions, and doping with other elements to improve the photocatalytic activity [103,104,105]. For example, Min Seok Koo et al. used electrochemical anodization to prepare vertically aligned TiO_2_ nanotubes (TNTs) then cathodically polarized them to obtain blue coloration TNTs (blue-TNTs) [21]. The dual-functional blue-TNT photoelectrode was employed for water treatment and H_2_ generation (Figure 8a). XPS results demonstrated that Ti^3+^ existed on blue-TNTs, which could adjust the absorption range of the solar spectrum. As a result, the diffuse reflectance spectroscopy of blue-TNTs (Figure 8b) showed a red shift and stronger UV absorption. In the pollutant degradation coupled with hydrogen production system, the defects in blue-TNTs improved the activity and stability. As shown in Figure 8c, the removal rate of 4-CP was 1.8 to 1.4 h^−1^ for blue-TNTs, which was more active than that of TNTs (0.40−0.25 h^−1^). And, the H_2_ generation rate was kept at 72−66 μM cm^−2^ h^−1^. In addition to building defects, the formation of heterojunctions could also broaden the visible light absorption range of TiO_2_. Cong et al. [106] prepared CdS/Ag/TiO_2_-NR ternary heterostructure catalysts for PEC oxidation of nitrobenzene (NB) with simultaneous H_2_ production (Figure 8d). From the UV-Vis diffuse reflectance spectra, it can be seen that the heterojunction catalysts were produced when Ag was combined with CdS/TiO_2_-NRs, and the absorption spectra of the catalysts were red-shifted due to the SPR effect of metal Ag combined with the n-type semiconductor TiO_2_. More photogenerated electrons could be generated through the SPR effect to further increase the electron transfer rate. In Figure 8e, the degradation rate of NB could be as high as 96% after 50 min of PEC activation. The composite catalyst reached 2.24 mmol of H_2_ yield with a yield of 0.09 mmol h^−1^ cm^−2^ under visible light irradiation for 6 h (Figure 8f).

Ta_3_N_5_, as an n-type semiconductor, is a promising photoanode material with the narrow bandgap of 2.1 eV [107,108]. It also owns a suitable redox potential band structure. However, it suffers from weak carrier transport and low photovoltage deficiencies. To improve the above deficiencies, the integration of the surface with water-oxidation co-catalysts enhanced the surface WOR kinetics as well as the facilitation of charge complexation and hole accumulation through the pore extraction and the injected electrolyte, and this was a method to significantly improve the Ta_3_N_5_ photoanode PEC activity and stability in an effective way. For instance, Shi et al. prepared a new Ta_3_N_5_-integrated photoanode modified by two-dimensional (2D) trimetal CoNiFe-LDHs NSs using a simple electrodeposition method [22], which was applied to the oxidation of glycerol for the production of formic acid and synchronized hydrogen production systems (Figure 8g). From the TEM, the catalysts were ultrathin nanosheets with rolled-up edges, indicating that they had abundant surface reaction sites (Figure 8h). From the Mott–Schottky plots, the 2D CoNiFe-LDH NSs could promote photogenerated carrier transfer and separation and significantly improve the performance of PEC. Nearly 100% FE of glycerol conversion to formate at the anode for 30 min and stable hydrogen production FE around 98% were achieved (Figure 8i,j), which could realize efficient and stable PEC hydrogen production as well as green synthesis of high-value chemicals through biomass conversion.

## 6. Conclusions and Future Challenges

In this review, different anodic oxidations (H_2_O_2_ oxidation, ·Cl oxidation, electroflocculation, SOR, ClOR, electrochemical oxidation of organics, etc.) were studied and summarized. Firstly, the electrocatalytic degradation of pollutants coupled with a hydrogen production system was summarized using different radical mediators (·OH, ·Cl) that play a major role in the degradation process in addition to the use of the electroflocculation method for pollutant degradation coupled with the hydrogen production system. Secondly, based on the upgrade of pollutants, the production of sulfur monomers and simultaneous hydrogen production in sulfur-containing wastewater systems, as well as the upgrade of organic pollutants in wastewater into high-value products, were realized. Finally, based on photoelectrocatalytic anodic oxidation coupled hydrogen production, the pollutants can be directly used as hole scavengers for photo-induced hydrogen production, which promotes the reaction and achieves the purpose of degradation.

Although great progress has been made in the electrolysis of wastewater for hydrogen production, there are still a lot of challenges in this area. The pollutants exhibit strong corrosiveness and volatility within the electrocatalytic system, which can affect the electrode’s lifetime and hydrogen purity. Moreover, the complexity of the actual pollutant components, each of which corresponds to a specific oxidation potential, can make the anodic degradation process more challenging in practical applications. Exploring efficient, stable, selective, and low-cost electrocatalysts for this coupled electrocatalytic strategy is a long-term challenge. In addition to this, for the organic upgrade of pollutants, it is only at the stage of small-molecule organics, and the research on large-molecule organic pollutant systems needs to be further improved. For PEC systems, improving the spectral absorption range of semiconductors is still a direction that needs unremitting efforts. With the development of catalysts and semiconductors, this emerging field will integrate redox reactions in electrolyzers and photoreactors, and there is still a lot of room for development in the future. In summary, the future of wastewater hydrogen production should achieve economic, environmental, and societal value. However, the composition of the actual wastewater is complex, and the composition is constantly changing. The currently reported hydrogen production from wastewater is simulated wastewater and only considers the reaction of pollutants and water while ignoring other cations (e.g., K^+^, Ca^2+^, Mg^2+^, Na^+^ etc.) and anions (e.g., F^−^, Cl^−^, Br^−^, PO_4_^3−^ etc.) present in the actual wastewater in which they may participate or affect the process of the reaction. So, the long-term stable production of hydrogen in actual wastewater is still challenging.

## Data Availability

Data are contained within the article.

## Abbreviations

OER	Oxygen evolution reaction
HER	Hydrogen evolution reaction
·OH	Hydroxyl radical
ROS	Reactive oxygen species
EPR	Electron paramagnetic resonance
·Cl	Chlorine radicals
SOR	Sulfion oxidation reaction
FE	Faradaic efficiency
ClOR	Chlorine electrooxidation reaction
OWS	Overall water-splitting
BHMF	2,5-bis(hydroxymethyl)furan
NF	Nickel foam
PEC	Photoelectrochemical
2D	Two-dimensional
